# Translation, transcultural adaptation and validation to Brazilian Portuguese of tools for adverse drug reaction assessment in children

**DOI:** 10.1186/s12874-021-01315-9

**Published:** 2021-07-08

**Authors:** Elisangela da Costa Lima, Thais de Barros Fernandes, Adair Freitas, Juliana Freire de Lima Sias, Marcelo Gerardin Poirot Land, Mariana Tschoepke Aires, Louise Bracken, Matthew Peak

**Affiliations:** 1 School of Pharmacy – Federal University of Rio de Janeiro, Rio de Janeiro, Brazil; 2 Instituto de Puericultura e Pediatria Martagão Gesteira- Federal University of Rio de Janeiro, Rio de Janeiro, Brazil; 3 Paediatric Medicines Research Unit, Alder Hey Children’s NHS Foundation Trust, Liverpool, UK

**Keywords:** Validation study, Translating, Adverse drug reaction reporting systems, Child

## Abstract

**Background:**

Children are more vulnerable to adverse drug reactions (ADRs) due to complex changes in the body during the growth process and lack specific pharmacoepidemiologic studies. Causality and Avoidability assessment of ADRs are relevant to clinical guidelines development and pharmacovigilance. This study aimed to translate and transcultural adapt two new tools—Liverpool Causality Assessment Tool (LCAT) and the Liverpool Avoidability Assessment Tool (LAAT)—to Brazilian-Portuguese and evaluate the psychometric properties of these tools to analyse ADRs in Brazilian children.

**Methods:**

The validation of the cross-cultural adaptation of tools was obtained by the functional (conceptual, semantic, operational, and measurement) equivalence between the original and translated versions of each instrument. The translated version of LCAT and LAAT was applied to assessing the twenty-six case reports of suspected adverse drug reactions in a Brazilian teaching paediatric hospital. The inter-rater reliability (a pharmacist and a physician) was evaluated using Cronbach’s alpha. The exact agreement percentages (%EA) and extreme disagreement (%ED) were computed. Overall Kappa index was calculated with a 95% confidence interval.

**Results:**

There was a need to modify some terms translated into Portuguese for semantic and conceptual equivalence. The Cronbach’s alpha coefficient values obtained were 0.95 and 0.85, and the weighted Kappa (95% confidence interval) were 0.82 (0.67–0.97) and 0.68 (0.45–0.91) for LCAT and LAAT, respectively. The Brazilian-Portuguese versions of the LCAT and LAAT showed reliable and valid tools for the diagnosis and follow-up of ADRs in children.

**Conclusion:**

The methodological approach allowed the translation, transcultural adaptation, and validation to Brazilian-Portuguese of two easy and quick to perform tools for causality and avoidability of ADRs in children by a multidisciplinary expert specialist committee, including the authors of original tools. We believe these versions may be applied by professionals (patient safety teams) and researchers in Brazil in groups or by a single reviewer.

**Trial registration:**

This study was evaluated and approved by the Research Ethics Committee (Instituto de Pediatria e Puericultura Martagão Gesteira – Federal University of Rio de Janeiro – Number: 3.264.238.

**Supplementary Information:**

The online version contains supplementary material available at 10.1186/s12874-021-01315-9.

## Introduction

Adverse drug reactions (ADRs) are harmful or unpleasant responses that occur unintentionally after using a drug. They may cause temporary or permanent physical damage, contributing to increased morbidity, mortality, and hospital admission costs [1, 2]. Children are more vulnerable to these reactions due to changes in the body resulting from the growth process and the scarcity of studies in this population [3]. In Brazil, almost two thousand suspected serious ADRs, including deaths, were reported in the National Health Surveillance Agency database (Notivisa) in children between 2008 and 2013 [4].

The causality (likelihood of a drug causing the ADR) and avoidability (possibility of ADR prevention) of an ADR are relevant to the study of drug safety. Such investigations may help develop protocols to aid prescribing, drug monitoring, and appropriate use of pharmacotherapy [5–7].

The most commonly used tools for assessment of the causality of ADRs, such as the algorithm proposed by Naranjo [8], have limitations for use in the paediatric population [9, 10]. The Naranjo tool, published in 1982, may have less sensitivity for use in children because the overall score obtained can be artificially reduced [11]. In the same way, the most commonly used scale for assessment of ADR avoidability, created by Hallas [12], calls for in-depth knowledge of treatment protocols used which often don’t exist for paediatrics [6, 13]. In Brazil, the Naranjo tool is frequently used in ADR causality assessment, although studies focused on children are scarce, notably, those who assess avoidability.

Because of these limitations, two English-specific tools for the characterisation of ADRs in children have been developed and validated: the Liverpool Causality Assessment Tool (LCAT) [11] and the Liverpool Avoidability Assessment Tool (LAAT) [14]. LCAT and LAAT tools are both flow diagrams which are quick and easy to use enabling a systematic approach to assessing causality and avoidability of ADRs in children. Both tools use dichotomous responses and were developed and validated using retrospective case reports in the United Kingdom [11, 14].

This study aimed to translate and transcultural adapt the LCAT and LAAT to Brazilian-Portuguese and to evaluate the psychometric properties of these tools for the analysis of ADRs in paediatric patients.

## Methodology

Translation and transcultural adaptation were performed in collaboration with the research group responsible for designing and validating the original English language versions of LCAT and LAAT. For an in-depth understanding of the construction and investigation of the psychometric properties of the tools, the corresponding author spent approximately 6 months based in the Paediatric Medicines Research Unit at Alder Hey Children’s Hospital (AHCH) (Liverpool, UK) for study design [15] and practical training.

**Fig. 1:**
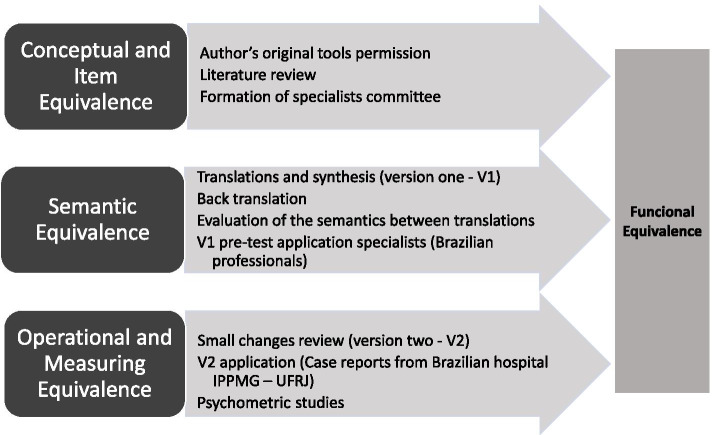
Main steps for translation and transcultural adaptation of LCAT and LAAT

The validation of the transcultural adaptation of tools was obtained by the functional equivalence (given by the degree of agreement between conceptual and item equivalence, semantic equivalence, operational and measurement equivalence) between the original versions and the translated versions of the tools (Fig. 1) [15, 16].

### Conceptual, item and semantic equivalence

A literature review was conducted to identify any new publications about the original instruments since their development and their use in the target population [16]. A committee of experts, made up of authors of the new (translated) version and the original version, assisted in identifying the concepts of terms and phrases used in the tools, considering the characteristics of the target population such as roles and health professionals’ practices [15].

The translation of LCAT and LAAT was performed independently by two professionals (a physician and a pharmacist) fluent in the source language and culture, considering the conceptual equivalence and avoiding the literal translation [15, 16]. Then, members of the committee of experts conducted a synthesis of translated texts in a single version (version 1—v1) of each tool and assessed any linguistic, conceptual, and contextual discrepancies [15, 16]. A retranslated version of each tool obtained from the source language (British English) from v1 was used to compare the original text’s content and evaluate semantic equivalence — meaning of the word or sentence in the Brazilian culture — and equivalence of items [17].

Application of v1 of each tool consisted of a pre-test performed by a group of 27 Brazilian professionals (24 pharmacists, 1 doctor, and 2 nurses with experience in pharmacovigilance). The objective was to verify the adequacy, structure, and usefulness of each of the tools. Any operational difficulties were recorded and discussed for each item of the tools [15]. Small changes made in response to points raised by the group resulted in version 2 (v2) of both tools.

### Operational and measuring equivalence

Martagão Gesteira Child Care and Paediatrics Institute of the Federal University of Rio de Janeiro (IPPMG—UFRJ, Rio de Janeiro, Brazil) was selected as the setting for the introduction of the versions of each tool translated and adapted into Brazilian Portuguese (v2). IPPMG—UFRJ is a medium-sized teaching hospital (80 beds) linked to the Single Health System (SUS) for highly complex paediatric care.

All suspected cases of ADRs related to anti-infectives in children aged 0 to 17 years hospitalized at IPPMG – UFRJ identified from an observational study with prospective data collection (between May and October 2019) were assessed with the exception of ADRs which occurred in the intensive care unit and oncology as these were excluded. Anti-infectives were chosen for this study because they were the main agents responsible for reports of ADRs in Brazilian children [18].

Information on; (a) patient characterization (age, gender, health, and medication history); (b) prescription of all drugs (including dosages), and; (c) details of suspected cases, was collected daily from patient diaries, laboratory results and medical records were used to prepare case reports [5, 6, 14]. The following were also considered: (i) pharmacology, (ii) known history of allergy or similar previous reaction, (iii) known preventative strategies, (iv) other sources of information or information in the history available for prevention of reactions; (v) appropriate measures taken to avoid an ADR, and, (vi) follow-up of ADR prevention strategies and management plan(s) [11, 14].

The v2 (in Brazilian-Portuguese) of both tools (LCAT and LAAT) were used to assess twenty-six cases from twenty-two children by two independent reviewers (a pharmacist and a paediatrician) who worked in the IPPMG—UFRJ. Prior to case assessment, the reviewers participated in a meeting (90 min) for structured discussion based on frequently asked questions about ADRs induced by anti-infectives in children and the use of the tools.

Each suspected ADR was evaluated for causality as unlikely, possible, probable, or definite, and for avoidability as unassessable, not avoidable, possibly avoidable, and definitely avoidable using the LCAT and LAAT, respectively. This measurement equivalence step was based on the following approaches of psychometric studies: (a) evaluation of dimensional validity and adequacy of component items, (b) reliability assessment, and (c) criterion validity and construct validity assessment [16]. It was considered the relevance and adequacy of each tool, the format of the questions/instructions, the way and scenario of application, and the categorization mode [16].

The inter-rater reliability was assessed using Cronbach’s alpha. The exact agreement percentages (%EA) were computed to measure the absolute concordances of the evaluator’s results. The %EA for each category was also estimated. The percentage of extreme disagreement (%ED) (in which the causality and avoidability scores between evaluators of the same case was greater than two) was measured between the pairs. This evaluation was based on an ordinal score of the outcomes for LCAT (unlikely = 0; possible = 1; probable = 2; definite = 3) and LAAT (unassessable = 0; not avoidable = 1; possibly avoidable = 2; definitely avoidable = 3 for LAAT), and it allowed %ED between raters to be estimated.

The overall Kappa index, which measures the nominal scale agreement among several raters, and linear weighted Kappa was calculated with 95% confidence intervals (CI) [11, 14, 19, 20]. Kappa values were interpreted according to the guidance from Altman [21]: poor < 0.2; fair 0.21 ± 0.4; moderate 0.41 ± 0.6; good 0.61 ± 0.8 and very good 0.81 ± 1 agreement. The statistical analysis was performed using IBM Statistical Package for the Social programs Statistics in Earth Sciences (SPSS) 19 and Microsoft Excel.

### Ethical issues

This study was evaluated and approved by the Research Ethics Committee (REC IPPMG—UFRJ – Number: 3.264.238.

## Results

The conceptual and semantic equivalence of items considered the different terms used by native translators. In addition, a grammatical review was conducted to ensure the correct use of the Brazilian-Portuguese language.

In the analysis of semantic equivalence with the group of 27 specialists, the format and ease of use of LCAT and LAAT were considered higher in comparison with other international algorithms, according to the previous experience of specialists. They highlighted the clarity of the technical terms and expressions used. However, it was suggested that the development of a supporting manual to aid in the application of the tools by inexperienced professionals would be beneficial.

For the LCAT, the main changes were related to the direct translation of questions and statements, including the word “drug”. There was also an exhaustive search for synonyms and the review of concepts related to the terms “lost-lasting disability” and “positive rechallenge” to improve the clarity of tools for the Portuguese language. For LAAT, the same was found for the terms “drug” and “medication”. The translation by the physician used the term “therapeutic conduct”. However, the literal translation was kept (due to the smallest number word count) (Table 1) to maintain the visual format of the English and Portuguese tools.

**Table 1:** Modified questions during semantic equivalence process of causality and avoidability tools

**Questions**	**Translation by native (Brazilian physician)**	**Translation by native (Brazilian pharmacist)**	**Version 1 (Synthesis)**	**Version 2 (post specialists’ application)**
**Causality Tool**				
Do you suspect an adverse drug reaction?	Você suspeita de uma reação adversa à droga?	Há suspeita de reação adversa a medicamento?	Há suspeita de reação adversa a medicamento?	Há suspeita de reação adversa a um medicamento?
Was the event associated with lost-lasting disability OR impairment?	O evento esteve associado a uma disfunção prolongada ou dano?	O evento foi associado a perda de capacidade duradoura ou dano?	O evento esteve associado a uma disfunção duradoura ou dano?	O evento esteve associado a perda de capacidade duradoura ou dano?
Was there a positive rechallenge?	Houve uma reexposição positiva?	Houve reexposição positiva?	Houve reexposição positiva?	Houve reexposição a uma dose subsequente com observação do mesmo evento?
**Avoidability Tool**				
Was there known preventable strategies and/or appropriate management plan (s), with information about ADR avoidance available?	Havia estratégias de prevenção conhecidas e/ou condutas terapêuticas apropriadas, com disponibilização de informações para evitar a RAM?	Havia estratégias conhecidas para a prevenção ou plano de manejo apropriado com informações sobre a prevenção da RAM?	Havia estratégias conhecidas para a prevenção ou plano de manejo apropriado com informações sobre a prevenção da RAM?	Havia estratégias conhecidas para a prevenção ou plano de manejo apropriado contendo informações sobre como evitar a RAM?
Were other information sources, or information in the history available for prevention of the ADR which could have been followed?	Outras fontes de informação, ou informações no prontuário, estavam disponíveis para a prevenção da RAM que poderiam ter sido seguidas?	Foram consideradas as fontes de informação ou a informação sobre a história do caso que pudessem ser seguidas?	Outras fontes de informação ou informações no prontuário que poderiam ter sido seguidas estavam disponíveis?	Outras fontes de informação que poderiam ter sido utilizadas para evitar a RAM estavam disponíveis?

*RAM* Reação adversa a medicamento

**Fig. 2:**
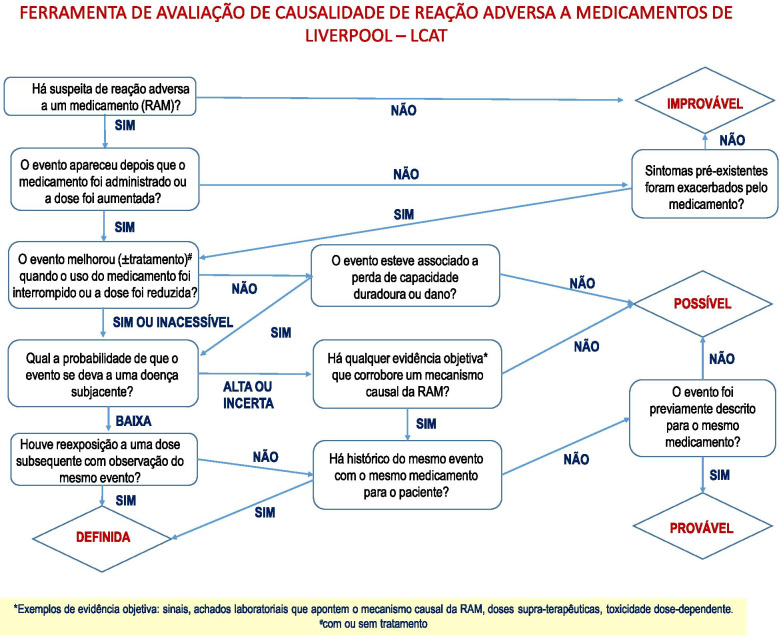
The translation of Liverpool Causality Assessment Tool for Portuguese

**Fig. 3:**
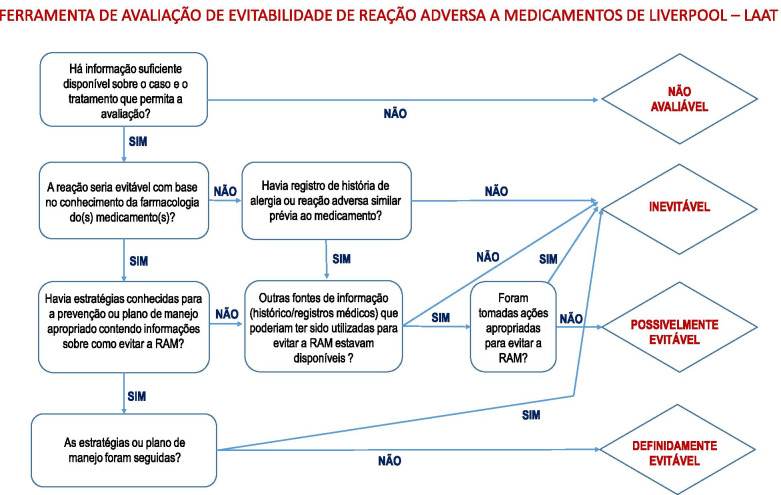
The translation of Liverpool Avoidability Assessment Tool for Portuguese

Version 2 of the tools (Figs. 2 and 3) were applied to the assessment of the twenty-six case reports of suspected adverse drug reactions to anti-infectives used by twenty-two children from IPPMG—UFRJ (Table 2).

**Table 2:** Patient characterization (*n* = 22) and ADR reported (*n* = 26) (Brazil, 2019)

**Data reported (frequency)**	**N**
**Age (years-old)**	
0 < 2	8
2 < 6	8
6 < 12	3
12 – 18	3
**Sex**	
Female	13
Male	9
**Diagnosis (health conditions)**	
Pneumonia	10
Other respiratory infections	2
Cellulitis	2
Mastocytosis	1
Multidrug-resistant tuberculosis	1
Pyelonephritis	1
Vasculopathy	1
Congenital syphilis	1
Acute gastroenteritis	1
Appendicitis	1
Short bowel syndrome and polyarthritis	1
**ADR reported**	
Nausea and Vomiting	9
Diarrhea	7
Pruritus, eczema or rash	3
Diaper rash	2
Anaphylaxis	1
Red man syndrome	1
Drug reaction with eosinophilia and systemic symptoms (DRESS)	1
Constipation	1
Dry cough	1

Cefepime, vancomycin, amoxicillin, amoxicillin clavulanate, azithromycin, fluconazole, amikacin, ampicillin, cefuroxime, oxacillin, gentamicin, linezolid, crystalline penicillin and trimethoprim-sulfamethoxazole were the anti-infectives identified in case-reports.

The Cronbach’s alpha coefficient, Cohen’s Kappa, Weighted Kappa, percentage of exact agreement (%EA) and extreme disagreement (%ED) between reviewers using LCAT and LATT v2 are presented in Table 3. The %EA for each category of tool as present in Table 4.

**Table 3:** Measurement equivalence results of Brazilian-Portuguese versions of tools

**Scale**	**LCAT (95% CI)**	**LAAT (95% CI)**
Cronbach’s alpha Coefficient	0.95 (0.89–0.98)	0.85 (0.71–0.93)
Cohen’s Kappa	0.73 (0.52–0.94)	0.65 (0.41–0.89)
Weighted Kappa	0.82 (0.67–0.97)	0.68 (0.45–0.91)
% Exact Agreement	80.7 (65.62–95.92)	76.9 (60.73–93.12)
% Extreme Disagreement	0 (0–0)	3.8 (0–11.24)

*LCAT* Liverpool Causality Assessment Tool, *LAAT* Liverpool Assessment Avoidability Tool, *CI* Confidence Interval

**Table 4:** Percentage of exact agreement for each category of Brazilian-Portuguese versions of tools

**Tool and Category**	**Assessor**	**Assessment**			**%EA**	**95% CI**	**% Standard Error**
	1	Yes	No	Total			
	2						
LCAT							
Definite	Yes	5	0	5	96.15	88.76–100.00^a^	3.77
	No	1	20	21			
	Total	6	20	26			
Probable	Yes	6	4	10	84.62	70.75–98.48	7.08
	No	0	16	16			
	Total	6	20	26			
Possible	Yes	7	1	8	84.62	70.75–98.48	7.08
	No	3	15	18			
	Total	10	16	26			
Unlikely	Yes	3	0	3	96.15	88.76–100.00^a^	3.77
	No	1	22	23			
	Total	4	22	26			
LAAT							
Definitely avoidable	Yes	6	0	6	84.62	70.75–98.48	7.08
	No	4	16	20			
	Total	10	16	26			
Possibly avoidable	Yes	8	1	9	92.31	82.06–100.00^a^	5.23
	No	1	16	17			
	Total	9	17	26			
Unavoidable	Yes	6	2	8	88.46	76.18–100.00^a^	6.27
	No	1	17	18			
	Total	7	19	26			

*LCAT* Liverpool Causality Assessment Tool, *LAAT* Liverpool Assessment Avoidability Tool, *CI* Confidence Interval, *% EA* Percentage of Exact Agreement

^a^Upper bound to 100.00% applied to keep the logical coherence

## Discussion

This study’s methodological approach allowed the translation, transcultural adaptation, and validation to Brazilian-Portuguese of existing tools for ADR assessment in children. Using the translated versions of each of these tools, the Cronbach’s alpha coefficient value obtained was acceptable (above 0.7), and the kappa values showed a good (avoidability) and very good (causality) reliability [21, 22]. These results indicate that the translated versions of LCAT and LAAT are reliable and valid for assessing the causality of suspected ADRs associated with anti-infectives and whether they could be avoided based on current knowledge and best evidence available in paediatric patients, respectively [23].

Regarding the translation and transcultural adaptation, it is also essential to highlight some issues relevant to the semantic equivalence of tools. In the translated versions of LCAT and LAAT, the term “drug” has been replaced by “medicine” because, although there is no significant difference in English, in the Portuguese language, “drug” is commonly related to “substances of abuse” [24]. Some terms were maintained in their literal translation of LCAT as “lost-lasting disability OR impairment” and “causal ADR mechanism” as well as in LAAT as “Information available” and “management plan”. The word order in sentence formulation did not represent changes in its meaning. It was decided to keep the asterisks present in the original version of LCAT, with a detailed explanation to help understand the question.

Some technical terms in the pharmacovigilance field in the English language have been changed to facilitate understanding. “Positive rechallenge” (observation of the same signs and symptoms with the administration of the same drug and after the reaction suspected to have disappeared [25] was translated to “*reexposição positiva*” and modified to “*reexposição a uma dose subsequente*” in the LCAT, to maintain the notion of temporality in the analysis.

The term “avoidance” (LAAT) has two translations: “*evitar*” or “*prevenir*”; with the former preferred by the study team. In most cases, these terms are interchangeable as it is considered that if an event can be avoided in the absence of errors, then it can be prevented [26].

Two aspects should be pointed about ADR causality and avoidability assessment and these may have influenced the degree of agreement observed between reviewers. The first is the quality of the writing of the prospective case reports. The causality assessment pre-supposes the availability of necessary information, particularly about event time to onset, rechallenge, dechallenge, allergies, underlying diseases, and other confounding factors. It is essential to investigate and summarize in the case report, for example, all doses of suspected medicine administered, and whether or not there is any mention in the patient’s medical documentation about the occurrence of previous events to ensure a robust analysis [10, 11]. However, the completeness of the patient’s medical record may be insufficient. Thus, it is strongly recommended that the collection of information is prospective and daily, whenever possible.

The second aspect is the ability of reviewers to search, rank, and interpret scientific evidence and information related to treatment with anti-infectives in children. Moreover, the rapid training provided before the first assessment (to mitigate uncertainties and doubts raised by the team of 27 specialists in the previous phase) allowed discussion and consensus on possible limitations in the search, especially regarding the prevention of ADRs. These strategies may be crucial because each reviewer’s perception relies on their professional background and experience [11].

All assessed cases could be classified using LCAT into one of the four outcomes. Most ADRs were considered definite or probable. LCAT has advantages over Naranjo’s algorithm when rating the causality of ADRs in children [11]. Naranjo’s algorithm is a weighted scoring system tool in which some variables (temporality, rechallenge, and non-existence of confounding factors) have a higher weighting (higher possible item score) and potential influence on the total score than others [10, 11]. On the other hand, LCAT uses a system based on binary decisions with some of Naranjo’s questions combined. If there is a record in the case report about the appearance of the reaction after drug administration, dechallenge, and no alternative cause for the event, it is possible to define the reaction with LCAT in cases of positive rechallenge, or the same event reported in the patient history. In addition, information commonly available only in clinical trials as placebo use is not considered in LCAT. Drug toxicity level detection in the blood (therapeutic drug monitoring), another unreliable source of data, is modified and combined with questions related to any objective evidence of ADR for causality assessment in LCAT [11].

One limitation of the present study was the assessment only of ADRs induced by anti-infectives. However, the English versions of these tools have been used to assess other medical groups using the same process, and there is no apparent reason why this would not be the case for the translated versions. In any case, information availability should be considered and may influence the analysis, as discussed above. Besides that, given Brazil’s cultural diversity, it is recommended that the tools are used in other Brazilian centres to confirm validation [27].

LCAT and LAAT are two easy and quick tools to perform the characterization of ADRs in children. We have translated and transcultural adapted both tools into Brazilian-Portuguese with a careful methodology by a multidisciplinary expert specialist committee, including the authors of original tools [15, 26]. Only 5% of the Brazilian population affirm to know the English language [28]. Thus, this version may be applied by professionals (patient safety teams) and researchers in Brazil in groups or by a single reviewer [14].

It seems appropriate to assume that the availability of LCAT and LAAT tools in Brazilian Portuguese will lead to better care by improved characterization of the risks of ADRs to antimicrobials and better prevention and management practices and promoting organizational changes [14, 27].

## Conclusion

The methodological approach allowed the translation, transcultural adaptation, and validation to Brazilian-Portuguese of the two new tools (LCAT and LAAT) for ADRs assessment in children. The availability of the translated versions of these tools may motivate paediatric pharmacoepidemiologic studies and drug use safety in this population.

## Supplementary Information

**Additional file 1.** Evaluation of causality and avoidability assessment.

**Additional file 2.** Case reports of suspected adverse drug reactions (Original version - in Portuguese).

## Data Availability

The datasets used and analysed during the current study are available from the corresponding author on request.
