# Supplementary material for: Translation, transcultural adaptation and validation to Brazilian Portuguese of tools for adverse drug reaction assessment in children
Source: BMC Med Res Methodol. 2021 Jul 8;21:141. doi: 10.1186/s12874-021-01315-9 (PMC8265060; doi:10.1186/s12874-021-01315-9)
Supplement: Supplementary file 2 — Additional file 2. Case reports of suspected adverse drug reactions (Original version - in Portuguese). [file 12874_2021_1315_MOESM2_ESM.docx]

**Supplementary file**

**Translation, Transcultural Adaptation and Validation to Brazilian Portuguese of Tools for Adverse Drug Reaction Assessment in Children**

Elisangela da Costa Lima*^1^, Thais de Barros Fernandes^1^, Adair Freitas^2^, Juliana Freire de Lima Sias^1^, Marcelo Gerardin Poirot Land^2^, Mariana Tschoepke Aires^2^, Louise Bracken^3^, Matthew Peak^3^

* Correspondence: [eclima.ufrj@gmail.com](mailto:eclima.ufrj@gmail.com)

^1^School of Pharmacy – Federal University of Rio de Janeiro, Rio de Janeiro, Brazil

^2^Instituto de Puericultura e Pediatria Martagão Gesteira- Federal University of Rio de Janeiro, Rio de Janeiro, Brazil

^3^Paediatric Medicines Research Unit. Alder Hey Children’s NHS Foundation Trust, Liverpool, United Kingdom

**Additional file 2**

**CASE REPORT MODEL**

| **PATIENT DATA** | | | | | |
| --- | --- | --- | --- | --- | --- |
| **Number**: | **Birth:** | | | **Age**: | **Gender** ( ) F ( ) M |
| **Admission date** | | **Chemo Regimen Name**: | | | |
| **Diagnose:** | | | **Weight**: | **Height**: | **Body surface:** |

|  | | **SUSPECTED DRUGS** | | | | | |
| --- | --- | --- | --- | --- | --- | --- | --- |
| **Date** | **Chemotherapy** | | **Dose** | **Route** | **Freq** | **Time start** | **Duration** |
|  |  | |  |  |  |  |  |

* Freq: Frequency

| **ADR** | | | |
| --- | --- | --- | --- |
| **Type** |  | **Severity:** | |
| **Date/time start:** | | | **Date/time finish:** |
| **Description:** | | | |

| **CASE SUMMARY** |
| --- |
| Medical history: (diagnose, underlying diseases), the reason for admission (description including symptoms).  Drugs history: (dose, route, treatment period, and information about any previous adverse drug reaction).  Record if an antidote was used and when. Include any suspicion of the team.  In case of any data unavailable, report as “information about (…) no available in medical records or patient/guardian does not remember about (…)”. It is essential for the correct response (no versus not know) in the assessment. |

| **OTHERS DRUGS** | | | | |
| --- | --- | --- | --- | --- |
| **Date** | **Drug** | **Dose** | **Route** | **Time** |
|  |  |  |  |  |
